# Anaesthetists’ knowledge of airborne infections

**DOI:** 10.4102/sajid.v37i1.351

**Published:** 2022-05-30

**Authors:** Ahmed Elghobashy, Juan Scribante, Helen Perrie, Dorinka Nel

**Affiliations:** 1 Department of Anaesthesiology, Faculty of Health Sciences, University of the Witwatersrand, Johannesburg, South Africa

**Keywords:** airborne, anaesthetists, knowledge, infections, healthcare, perioperative

## Abstract

**Background:**

Anaesthetists need to be knowledgeable regarding the control of airborne infection to ensure safe practice. The aim of this study was to determine anaesthetists’ knowledge regarding airborne infections in the perioperative period in the Department of Anaesthesiology at the University of the Witwatersrand.

**Methods:**

A cross-sectional research design was followed using an anonymous self-administered questionnaire. Data were collected at academic departmental meetings by convenience sampling. Returning the questionnaire implied consent. A score of 65% was considered adequate knowledge.

**Results:**

Of the 150 questionnaires distributed, 137 (91.3%) questionnaires were returned. An overall mean (standard deviation [s.d.]) score of 58.8% (4.252) was achieved, and only 11 (8.1%) of anaesthetists had adequate knowledge. There was no statistically significant association between seniority and passing or failing (*p* = 0.327). The highest mean (s.d.) score, 67.4% (6.979), was reported in the section pertaining to patients, followed by the section regarding operating theatre staff at 58.1% (11.899) and the lowest mark, 53.5% (5.553), for the environment section. Anaesthetists scored significantly better in the knowledge regarding patients’ section than in other sections (*p* < 0.0005).

**Conclusion:**

Knowledge of airborne infections in this study was poor, with only 8.1% achieving a pass, and no difference in knowledge between junior and senior anaesthetists was observed. Considering the ongoing coronavirus disease 2019 (COVID-19) pandemic at the time of the study, this was a surprising finding. Urgent action needs to be taken to ensure the safety of anaesthetists, other operating theatre staff and patients.

## Background

Hospital-acquired infections result in increased morbidity and mortality in about 10% of surgical cases, resulting in an increase in the length of hospital stay, hospital re-admission rate and overall cost for surgical patients.^1^ Airborne infections are responsible for an estimated 10% of hospital infections.^2^ The global outbreak of the coronavirus disease 2019 (COVID-19) pandemic has highlighted the importance of the knowledge of prevention and management of airborne infections among healthcare workers.

A variety of microorganisms, including viruses, bacteria and fungi, are known to be airborne. Examples include Mycobacterium tuberculosis, influenza-A viruses, varicella-zoster, rubulavirus, measles, Bordetella pertussis, Streptococcus pneumoniae and Mycoplasma pneumoniae.^3^ Other highly virulent respiratory pathogens include multidrug- and extreme drug-resistant Mycobacterium tuberculosis, severe acute respiratory syndrome coronavirus 1 (SARS-CoV-1), H1N1 pandemic influenza, H5N1 avian influenza, smallpox and polio,^4^ and more recently, SARS-CoV-2.^5,6^ The World Health Organisation estimated almost half a million active tuberculosis cases in South Africa in 2015 and estimated a further 1% of the population developing active tuberculosis every year.^7^ Influenza A was responsible for 17 000 deaths worldwide in 2009–2010, while SARS spread to more than 35 countries in 2002–2003, costing the world $18 billion.^8^ COVID-19 was responsible for 1.4 million deaths worldwide in the first 18 months of the pandemic.^9^

Patients are not the only ones at risk of infections. Healthcare workers also need to protect themselves against these microorganisms.^10^ In order to reduce the risk of exposure of healthcare workers and other patients, appropriate precautions should be in place to prevent and control airborne infections.^11^ An operating theatre has a high volume of patients with a fast turnover. It also has a high number of healthcare workers consisting of surgeons, anaesthetists, nursing staff, cleaners, porters and others. If these individuals are infected with airborne diseases, there is a risk for others sharing the space.

Ideally, the operating theatre environment should be free of pathogenic microorganisms, but this is an unrealistic goal. Alternatively, the risk of transmission of microorganisms between patients and healthcare workers should be minimised. This could be achieved through adherence to strict infection prevention and control principles, especially airborne precautions.^11^ Adherence to these principles appears to be suboptimal.^12^ To adhere to these principles, healthcare workers need to be knowledgeable regarding these microorganisms, how they are transmitted and how the perioperative environment can be manipulated to decrease the risk of transmission. This study aimed to determine anaesthetists’ knowledge regarding airborne infections in the perioperative period working in the Department of Anaesthesiology at the University of the Witwatersrand (WITS).

## Methodology

The study population comprised anaesthetists (medical officers, registrars and consultants) working in the Department of Anaesthesiology. The department consisted of 22 medical officers, 112 registrars and 74 consultants. A convenience sampling method was used, and questionnaires were administered to the entire accessible population. A minimum response rate of 60%, from 125 anaesthetists, was considered acceptable.^13^ Junior anaesthetists were defined as medical officers and registrars with ≤ 3 years of training and senior anaesthetists as registrars with ≥ 4 years of training and consultants.

No suitable questionnaires pertaining to anaesthetists’ knowledge regarding airborne infections could be identified. Following a review of the literature, a draft questionnaire was compiled, which ensured content validity. The draft questionnaire was reviewed by three anaesthesiologists, two with an interest in infection control and one in medical education, thereby ensuring face and content validity. The anaesthesiologists’ recommendations were incorporated into the final questionnaire. The questionnaire consisted of a demographic and three knowledge sections. The three knowledge sections contained nine questions pertaining to the patient, five questions pertaining to the operating theatre staff and 14 questions pertaining to the perioperative environment.

Data were collected at departmental academic meetings. One author (A.E.) was present, while the questionnaires were completed to address any queries and prevent data contamination. Anaesthetists were requested to return all questionnaires folded, whether completed or not, into a sealed box at the exit of the venue.

Blank questionnaires were used to calculate the response rate but thereafter were excluded. Incomplete questionnaires were included in the study, and knowledge questions not answered were considered incorrect. The questions were multiple choice, with each question having four choices. Some questions had more than one correct answer. Returned questionnaires implied consent. Adequate knowledge (pass mark) was determined as 65% using the modified Angoff method.^14^

Data were analysed in consultation with a statistician using Statistical Package for Social Sciences (SPSS) (Statistics for Windows, version 25.0. Armonk, NY: IBM Corp.). Categorical data were described using frequencies and percentages, and continuous data were described using means and standard deviations. The knowledge between junior and senior anaesthetists, overall and for the three knowledge sections, was compared using independent t-tests. The association between being junior or senior and passing or failing the questionnaire was analysed using the chi-square test. The difference in knowledge between the three sections was determined using repeated-measures analysis of variance (ANOVA) with a post hoc Bonferroni correction. A *p*-value of < 0.05 was considered statistically significant.

### Ethical considerations

Approval to conduct the study was obtained from the Human Research Ethics Committee (Medical) (M200155) at the University of the Witwatersrand and other relevant authorities. A cross-sectional research design was followed.

## Results

Of the 150 questionnaires distributed, 137 (91.3%) were returned, representing 65.9% of anaesthetists in the department. The characteristics of the anaesthetists are shown in Table 1. There were 67 (48.9%) junior and 69 (50.4%) senior anaesthetists.

**TABLE 1:** Characteristics of anaesthetists.

Characteristic	Number	Percent
**Professional designation**		
Medical officer	28	20.4
Registrar (< 3 years training)	39	28.5
Registrar (≥ 3 years training)	27	19.7
Consultant	42	30.7
Missing data	1	0.7
**Sex**		
Male	53	38.7
Female	79	57.7
Missing data	5	3.6
**Years of experience**		
0–5	71	51.8
6–10	35	25.5
11–15	14	10.2
> 15	16	11.7
Missing data	1	0.7

Note: Only 11 (8.1%) anaesthetists achieved a pass score for the questionnaire, with four (36.4%) being junior anaesthetists and seven (63.6%) being seniors. There was no significant association between seniority and passing or failing (*p* = 0.372).

Anaesthetists’ overall knowledge of airborne infections as well as the knowledge per section: patient, theatre staff and environment, is shown in Table 2. There was a significant difference between the knowledge in all three sections (*p* < 0.0005). The knowledge of patient-related factors was significantly better than that about theatre staff (*p* < 0.0005) and that about the environment-related factors (*p* < 0.0005), and the knowledge about theatre staff factors was significantly better than that of the environment-related factors (*p* < 0.0005).

**TABLE 2:** Anaesthetists’ knowledge of airborne infections.

Section	Knowledge scores (%)			
	Mean	s.d.	Minimum	Maximum
Overall	58.8	4.252	49.1	67.0
Patient	67.4	6.979	50.0	86.1
Theatre staff	58.1	11.899	30.0	90.0
Environment	53.5	5.553	39.3	66.1

s.d., standard deviation.

**FIGURE 1:**
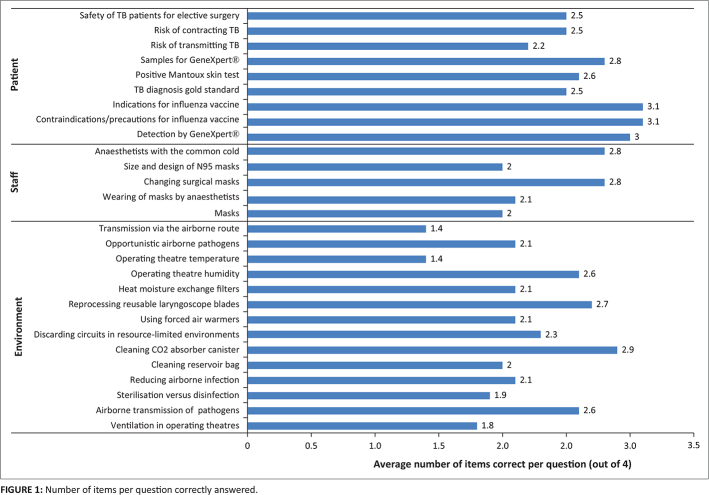
Number of items per question correctly answered.

Note: The average number of the four items per question that were answered correctly is shown in Figure 1.

A comparison between junior and senior anaesthetists’ overall knowledge of airborne infections as well as the knowledge per section: patient, theatre staff and environment is shown in Table 3. There were no significant differences.

**TABLE 3:** Comparison between junior and senior anaesthetist knowledge.

Section	Knowledge scores (%)		*p*
	Mean	s.d.	
**Overall**			0.997
Junior	58.8	4.031	
Senior	58.8	4.505	
**Patient**			0.646
Junior	67.7	7.442	
Senior	67.1	6.583	
**Theatre staff**			0.879
Junior	58.3	12.357	
Senior	58.0	11.610	
**Environment**			0.622
Junior	53.3	5.129	
Senior	53.8	5.998	

s.d., standard deviation.

## Discussion

The overall score obtained in this study was 58.8%, with only 11 anaesthetists achieving a pass mark of 65% or above. No previous studies specific to knowledge of healthcare workers on airborne infections could be identified. Several studies on the knowledge and practice of anaesthetists and other healthcare workers of infection control, in general, with a brief mention of airborne infections have been conducted. Some of these studies showed similar poor knowledge. Singh et al.^15^ concluded that dental students in India had low-to-average knowledge of droplet and airborne isolation precautions. In a study on aspects of occupational health that included airborne infections, Kim et al.^16^ in Brazil reported that the anaesthetists’ knowledge did not meet the expected levels. Other studies showed better knowledge but poor practice. Halboub et al.^17^ concluded poor compliance with infection control practices despite having acceptable knowledge amongst dental students at a university in Yemen. Nzioka^12^ at the University of Nairobi, Kenya reported similar results amongst anaesthetists regarding infection control practices.

Many of the items tested in this study are addressed in the Society of Anaesthesiologists of South Africa Infection Control Guidelines^18^ that guide the practice of South African anaesthetists. This study was planned prior to the COVID-19 pandemic; however, data were collected during the pandemic. The authors assumed that with the increasing awareness of airborne infections at this time, the results would be influenced. Therefore, it was surprising that despite all the available information, this did not translate into adequate knowledge.

A study assessing the knowledge, attitudes and practice of anaesthetists in preventing COVID-19 spread in Ghana^19^ concluded that adequate knowledge did not always translate to satisfactory attitude and practice. Chan et al.^20^ examined the relationship of knowledge, attitudes and practice of operating room staff on implementing standard- and transmission-based precautions using a two-step cluster analysis. Two clusters were identified. The authors found that the cluster with good knowledge, practice and a positive attitude implemented standard- and transmission-based precautions better than the cluster with poor knowledge, practice and attitude.^20^

There was no association between the seniority of anaesthetists and adequate knowledge of airborne infections. Senior and junior anaesthetists scored similarly in the questionnaire overall and in all the three sections. Kim et al.^16^ evaluated anaesthesiologists’ knowledge regarding occupational health and also found no significant difference in knowledge between senior and junior anaesthetists when asked about personal protective equipment (PPE) for droplet isolation precautions.

In this study, anaesthetists scored the highest (67.4%) in the section that focused on preventing and managing airborne infections in patients. A possible explanation is that prevention and management of airborne infections form part of the undergraduate curriculum and are also attained from clinical experience. The lowest score (53.5%) was in the perioperative environment section focusing on airborne dynamics and management and care of the operating theatre and equipment in terms of dealing with airborne infections. It is possible that anaesthetists may not be aware that current common practices are not in line with infection prevention and control principles. Adequate knowledge would empower anaesthetists to collaborate with other departments such as the department of infrastructure development, maintenance and the theatre management teams.

Regarding wearing masks during airway instrumentation, 53% of anaesthetists did not think that this was necessary. This was surprising as it speaks to anaesthetists’ self-protection. In a United Kingdom study of first responders’ knowledge of PPE requirements during the severe acute respiratory syndrome epidemic, anaesthesiology registrars achieved a score of 76%.^21^ Surgical masks should be worn during procedures with the potential to generate respiratory droplets, such as performing cardiopulmonary resuscitation and airway management.^11^ However, where there is a risk of transmission of airborne pathogens during airway management, an N95 mask as part of PPE should be worn.^22^

The South African Society of Anaesthesiologists (SASA) Infection Control Guidelines^18^ state that breathing circuits should be discarded after 7 days. However, only 23.3% of anaesthetists in this study answered this question correctly. This low number could be because of anaesthetists being unfamiliar with the SASA Infection Control guidelines.^18^ A further explanation could be that at the WITS affiliated hospitals, anaesthetic nurses are responsible for the management and care of breathing circuits, and it is assumed that they are correctly managed.

The SASA practice guidelines^23^ state that general operating theatre temperatures should range between 20°C and 23°C. Of the anaesthetists, only 35% answered this question correctly in this study. This result cannot be explained as anaesthetists at the WITS affiliated hospitals make decisions daily for surgery to proceed based on the operating theatre temperatures.

The study was carried out contextually in the Department of Anaesthesiology at WITS; therefore, the results may not be generalised to other institutes. The authors recommend that regarding airborne infections, the SASA Practice^23^ and Infection Control^18^ Guidelines should be incorporated in the Department’s standard operating procedures, and regular audits should be conducted to ensure compliance. Furthermore, the prevention of airborne infections should be included in the registrar curriculum and departmental in-service training.

## Conclusion

The knowledge of airborne infections among anaesthetists in this study was poor, with only 8.1% achieving a pass mark and no difference in knowledge between junior and senior anaesthetists. Considering the ongoing COVID-19 pandemic at the time of the study, this was a surprising finding. Urgent action needs to be taken to ensure the safety of anaesthetists, other operating theatre staff and patients.
