# Erratum: Anaesthetists’ knowledge of airborne infections

**DOI:** 10.4102/sajid.v37i1.456

**Published:** 2022-08-31

**Authors:** Ahmed Elghobashy, Juan Scribante, Helen Perrie, Dorinka Nel

**Affiliations:** 1 Department of Anaesthesiology, Faculty of Health Sciences, University of the Witwatersrand, Johannesburg, South Africa; 2 Surgeons for Little Lives, Department of Paediatric Surgery, School of Clinical Medicine, Faculty of Health Sciences, University of the Witwatersrand, Johannesburg, South Africa

In the published article, Elghobashy A, Scribante J, Perrie H, Nel D. Anaesthetists’ knowledge of airborne infections S Afr J Infect Dis. 2022;37(1):a351. https://doi.org/10.4102/sajid.v37i1.351, there was an error in the affiliation for the second author Juan Scribante. Instead of ‘Department of Anaesthesiology, Faculty of Health Sciences, University of the Witwatersrand, Johannesburg, South Africa’ it should be ‘Surgeons for Little Lives, Department of Paediatric Surgery, School of Clinical Medicine, Faculty of Health Sciences, University of the Witwatersrand, Johannesburg, South Africa’.

The publisher apologises for this error. The correction does not change the study’s findings of significance or overall interpretation of the study’s results or the scientific conclusions of the article in any way.

